# Supplementary material for: Are carbon emissions trading and green financial instruments synergistic? -Comprehensive quantitative research based on content analysis
Source: PLoS One. 2024 Mar 7;19(3):e0298601. doi: 10.1371/journal.pone.0298601 (PMC10919601; doi:10.1371/journal.pone.0298601)
Supplement: S1 Data — (ZIP) [file pone.0298601.s001.zip › Data/地级市Z_F工作报告.zip!/╕╩╦α/╬Σ═■╩╨/└┤╫╘╣1⁄2╓┌║┼í╛┬φ┐╦╩2╛▌═°í┐_2017.docx]

各位代表：

现在，我代表市人民政府向大会作工作报告，请予审议，并请市政协委员和列席人员提出意见。

过去五年的工作

市第三届人民代表大会第一次会议以来的五年，是全市发展进程中很不平凡的五年。面对艰巨繁重的改革发展稳定任务，我们在省委、省政府和市委的坚强领导下，全面贯彻党的十八大和十八届三中、四中、五中全会精神，深入学习贯彻习近平总书记系列重要讲话精神和治国理政新理念新思想新战略，紧盯脱贫攻坚、全面建成小康社会奋斗目标，树立以党建统揽全局、用发展凝聚人心，无中生有抓项目、有中生优增效益，严格标准选干部、不拘一格用人才的理念，竭力实施开放外向、工业强市、城乡融合、生态立市战略，改革创新，真抓实干，奋力推动科学发展、转型升级、富民强市，圆满完成“十二五”规划目标任务，开创了武威经济社会发展新局面。

——综合实力迈上新台阶。预计到2016年底，实现生产总值456亿元，是2011年的1.7倍，年均增长10.9%；人均生产总值由2011年的15007元增加到25121元，年均增长10.9%。全社会固定资产投资935亿元、增长13%，其中规模以上682亿元、增长10%，五年累计完成全社会固定资产投资3678亿元、年均增长25.2%，其中规模以上2813亿元、年均增长19%。实施招商引资项目2546项，落实到位资金3759亿元，是上个五年的19.5倍，年均增长60%。全部工业增加值107.2亿元，是2011年的1.3倍，年均增长13%，其中规模以上88.5亿元，是2011年的1.5倍，年均增长12.7%。大口径财政收入50亿元，是2011年的2.5倍，年均增长19.9%；一般公共预算收入30.9亿元，是2011年的3倍，年均增长24.9%；一般公共预算支出187.9亿元，是2011年的1.9倍，年均增长14.1%。城镇居民人均可支配收入23650元，是2011年的1.8倍，年均增长12.3%；农村居民人均可支配收入10100元，是2011年的1.9倍，年均增长14.2%。居民消费价格指数涨幅控制在3%以内。到今年9月末，金融机构存贷款余额分别达到812.9亿元、738.4亿元，是2011年底的2.1倍、3.6倍。

——脱贫攻坚取得新成效。贫困区生产生活条件明显改善，产业基础基本形成，群众收入持续增加。贫困人口由2011年的56.07万人预计减少到2016年底的4.33万人，贫困面由35.44%下降到2.9%，民勤县整体脱贫，凉州区可在年底整体脱贫。坚持实施“下山入川”工程，建成古浪黄花滩、天祝南阳山片等生态移民安置区，祖祖辈辈生活在高深山区的贫困农牧民移居川区、拔除穷根。五年累计移民28919户、110709人。贫困户累计发展设施农牧业20.9万亩、特色林果业31万亩。硬化贫困村道路1336公里，建成汽车停靠点309个，新建、改建学校（幼儿园）112所，建成标准化卫生室522个、综合性文化服务中心（乡村舞台）275个，实现动力电全覆盖，解决了25.8万贫困人口安全饮水问题。五年累计投入财政专项扶贫资金33.9亿元。

——产业发展形成新格局。新型工业体系初步建立。累计完成工业投资1211亿元，是上个五年的4.6倍，年均增长18.1%。着力培育产业链经济，中国航天武威新能源装备制造产业园、甘肃建投新能源装备制造产业园、青啤28万千升啤酒、京奥港30万吨高档饮品、承威专用车、凯帝斯电梯、荣华易地搬迁循环经济技术改造、鑫淼电石深加工、祁连山水泥等一大批骨干工业项目建成投产，达利、莫高等一批骨干企业达产达标。风光电装机规模达到300万千瓦。工业园区建成面积99平方公里，入驻企业605户，分别是2011年的2.1倍和1.7倍。省、市级工程技术研究中心、企业技术中心、高新技术企业分别达到35家、53家和13家。实施入园工业项目和“小升规”企业固定资产贷款贴息奖补、小微企业互助贷款风险补偿担保基金等政策，推进重点项目互保共建、工业企业互为市场。发展循环经济，加强节能减排，单位工业增加值能耗、用水量累计分别下降32.6%和24.5%，我市被列为全省循环经济试点城市、创建国家循环经济示范城市。

坚持以持续增加农民收入为核心，加快调整农业产业结构，强力推进“设施农牧业+特色林果业”主体生产模式。五年新增设施农牧业58.7万亩，累计达到90万亩，户均2.6亩；新增特色林果基地137.1万亩，累计达到168.5万亩，人均1.1亩。设施瓜菜、肉类和特色林果产量分别达到131.5万吨、19万吨和23.3万吨，农业增加值由2011年的67亿元预计提高到108亿元，农村居民人均可支配收入60%来自主体生产模式。实施50项出口农产品生产技术规程，建成出口农产品基地65万亩，辐射带动周边区域120万亩。认证“三品一标”农产品161个。大力推行“贮藏加工+运输销售”现代营销模式，市级以上农业产业化龙头企业达到146家，农民专业合作社8100家，设施农产品外销量占总产量的70%以上，冷藏储备库容达到37万吨。累计种植甜高粱85.3万亩。

现代服务业加快发展。新建、续建生活消费品、生产资料等各类市场93个，建成农家店1263个、商品配送中心14个、商贸中心4个。万嘉国际广场、新圣园家居汇展中心等一批大市场建成运营，红星时代广场、万嘉汇•台湾士林不夜城、武威天马中心等项目加快建设。电子商务快速发展，2016年全市预计实现网络零售额25亿元以上。华夏文明传承创新区武威项目区加快建设，《金色大道•马踏飞燕大景区总体规划暨节点修建性详细规划》获批实施，莫高•中国葡萄酒城、塔儿湾祁连冰雪嘉年华滑雪场一期工程等项目建成投运。我市被命名为“中国葡萄酒城”，成功举办4届“中国•河西走廊有机葡萄美酒节”。

——开放外向实现新突破。围绕打造丝绸之路经济带黄金节点和全省外向型经济发展龙头，举全省之力建设甘肃（武威）国际陆港。《甘肃（武威）国际陆港发展战略规划（2016—2030）》获省政府批复实施。甘肃首个海关特殊监管区—武威保税物流中心封关运行，中欧国际货运班列“天马号”累计发运51列、货运量7.5万吨、货运总值1.57亿美元。武威进境木材集中监管区、进口肉类查验场获批建设，陆港基础设施建设加快推进。武威天马机场列入国家民航总局“十三五”民航发展规划，武威铁路口岸列入甘肃省“十三五”口岸发展规划。甘肃路斯宠物食品等16个项目入驻陆港，跨境电商全球直购体验中心建成运营，武威众兴菌业循环经济示范园开工建设。预计全市进出口总额由2011年的0.85亿元增加到4.1亿元。

——城乡融合开创新局面。坚持以城乡融合发展核心区建设带动城乡全面发展，金色大道区域产业集聚效应凸现，资源共享快速推进，民勤、天祝县域核心区集聚带动能力增强，民勤县和凉州区黄羊镇、古浪县黄羊川镇新型城镇化试点有序推进。凉州城区建成金色大道城区连接线、滨河东路等55条主次道路，地表水供水工程、污水再生利用工程建成投运，天然气综合利用、城区集中供热等项目加快实施。体育馆建成投用，博物馆、影剧院等公共设施基本建成。天马湖景观治理工程全面建成。实施祁连大道、学府路及新老城区过渡区域市容环境综合整治。全市城镇建成区面积新增34平方公里，达到101.9平方公里，城镇化率由2011年的28.9%提高到38.8%。创建省级“千村美丽”示范村24个、市县区级80个，“万村整洁”村469个。

——生态环境建设取得新成果。石羊河流域重点治理任务全面完成，蔡旗断面过水量和民勤盆地地下水开采量两大约束性指标、生态治理目标分别提前8年、6年实现。干涸51年的青土湖重现水面25.2平方公里，地下水埋深由2011年的3.6米升至3.04米，形成旱区湿地106平方公里，局地小气候有效改善。祁连山水源涵养林及高山湿地、旱区湿地加快恢复，湿地总面积达到1042平方公里，居全省市州第4位。甘肃内蒙省界武威段千里沙漠大林带加快建设。加强“下山入川”移民迁出区生态恢复治理，依法清理整治祁连山国家级自然保护区内建设项目。坚持干部义务压沙制度。严格落实“五禁”决定。累计完成人工造林259.6万亩、封育185.5万亩，通道绿化5648公里，森林覆盖率由2011年的12.1%提高到19.7%。全力打好凉州城区大气污染防治攻坚战，大气环境质量持续改善。我市被命名为全国节水型社会建设示范区、防沙治沙综合示范区。

——基础设施得到新改善。永（登）古（浪）、营（盘水）双（塔）、金（昌）武（威）、民（勤）红（砂岗）高速和金色大道、天（祝）互（助）二级公路建成通车，北（山）仙（米寺）高速公路民勤县至凉州区段及绕城高速加快推进，大靖至干城、凉州至古浪等公路开工建设。新增高等级公路843.6公里，其中高速公路470公里。新建、改建农村公路6806公里。公路总里程达到1.34万公里，路网密度达到40.4公里/百平方公里。干（塘）武（威）铁路二线建成。建成古浪黄花滩水利骨干工程、天祝南阳山片“下山入川”生态移民小康供水工程、杂木河毛藏寺水库等重点水利工程，红崖山水库由中型扩建为大型工程。甘电投武威热电联产项目进展顺利。新建330千伏变电站3座，新建、扩建110千伏变电站9座，电网保障能力明显增强。

——民生保障有了新提升。累计新增城镇就业14.5万人，城镇登记失业率控制在4%以内。建成保障性住房12.3万套、完成棚户区改造1.9万户，完成农村新房建设7万户、危房改建7.8万户，建成新农村社区126个。解决了80.2万农村人口、15.5万农村师生的饮水安全问题。城镇居民基本医疗保险制度全面实施，城乡居民大病医疗保险工作全面启动，新型农村社会养老实现全覆盖，新农合参合率、城乡居民养老保险参保率、城乡低保和农村五保供养标准连续提高。集中供养“三无”残疾人670人。民勤县、天祝县、凉州区通过义务教育发展基本均衡县国家评估认定。九年义务教育巩固率、高中阶段教育毛入学率分别达到99.4%、92.5%。武威职业学院成功创建成国家骨干高职院校，兴办兰州交通大学新能源与动力工程学院、甘肃威龙葡萄酒业专修学院。中国首台自主研发的医用重离子加速器建成，市人民医院整体搬迁新建，恶性肿瘤高发区防控模式示范项目全面完成，乙肝防治示范区建设项目持续推进。建成乡村综合性文化服务中心（乡村舞台）940个、乡镇及社区体育健身中心和村级体育场770个。省、市列民生实事全面完成。

——深化改革取得新进展。大力简政放权，承接省级取消下放调整行政审批项目335项，精简市级行政审批项目426项。编制市、县区政府部门权责清单并向社会公布。建成全市统一公共资源交易平台。供给侧结构性改革加快推进。“营改增”试点全面推开。商事制度改革逐步深化。鼓励支持非公经济发展，非公经济增加值占生产总值比重达到65%。创新融资方式，增加信贷投入，连续3年获得“省长金融奖”。把普惠政策转化为特殊政策，累计发放“妇小贷”“双联贷”“双业贷”和精准扶贫、易地扶贫搬迁等五项惠农贴息贷款303亿元，户均8.8万元，覆盖面80%，各级财政贴息25亿元。组建武威农商银行、民勤农商银行，设立甘肃银行、兰州银行、交通银行分支机构，农村便民金融网点增加到2121个。成功发行企业债、私募债等直接融资45.86亿元。设立工业发展财政引导基金和现代农业创投基金。运用政府和社会资本合作（PPP）模式推进北（山）仙（米寺）高速公路等重大项目建设。农业农村综合改革和水权水价改革稳步实施，农村产权确权颁证和抵押贷款工作扎实推进。农村土地流转84.8万亩，土地规模经营27万亩，治沙生态林承包经营180.9万亩。坚持“管好用活”土地，完善土地统筹使用机制，重点产业、重大项目和民生工程用地保障有力。将凉州城区土地、建设管理、道路运输管理权由市级下放凉州区，完成食品药品、工商、质监、公安交警管理体制和公务用车改革，供销合作社综合改革试点、国有企业改革积极推进。省级创新型试点城市建设通过评估考核。

——政府自身建设得到新加强。坚决贯彻中央八项规定和省委、市委“双十条”规定精神，扎实开展党的群众路线教育实践活动、“三严三实”专题教育和“两学一做”学习教育，严管干部、善待群众，持续整治贪假懒庸、为官不为，高度警惕伪善伪廉伪勤问题，干部作风明显好转。认真学习贯彻《中国共产党廉洁自律准则》《中国共产党纪律处分条例》《中国共产党问责条例》和省委《实施办法》，严格落实党风廉政建设主体责任体系，廉政建设不断加强。坚持零容忍、下狠手，严肃查处违纪违法案件，保持惩治腐败的高压态势。全面落实市委决策部署，认真执行市人大决议，充分吸纳市政协意见。坚持依法行政，完善政府工作规则，建立政府重大行政决策合法性审查机制，法治政府建设取得新进展。创新社会治理，开展重大事项社会稳定风险评估，预防化解矛盾纠纷和信访突出问题。严格落实安全生产责任制，安全生产形势稳定好转。全面贯彻落实党的民族政策和宗教工作方针，民族团结，宗教和顺，社会大局和谐稳定。国防动员和“双拥”工作进一步加强，连续3年荣获“全国双拥模范城”。统计、信访、气象、人防、消防、邮政、老龄、外事侨务、防震减灾、档案、地方志、妇女儿童和慈善事业等工作取得新成绩。

各位代表，过去五年是我们砥砺前行、奋力拼搏、勇于作为的五年，是武威改革力度最大、项目建设最多、投资规模最大、发展速度最快、综合效益最好、基础设施和生态环境改善最明显、城乡面貌变化最显著、社会治理成效最突出、人民群众得到实惠最多的五年。五年发展的巨大成就，是省委、省政府和市委正确领导的结果，是市人大、市政协和社会各界同心协力、监督支持的结果，是全市各族人民锐意进取、团结奋斗的结果。我代表市人民政府，向全市各族人民，向各民主党派、工商联、人民团体和社会各界人士、离退休老同志，向驻武各部队、武警官兵、民兵预备役人员，向中央、省属在武各单位以及所有积极参与、关心支持武威改革发展稳定的同志们、朋友们，表示衷心感谢和崇高敬意！

五年来，我们战胜了困难挑战，积累了实践经验。必须坚持党的领导，坚定正确政治方向，牢固树立政治意识、大局意识、核心意识、看齐意识，坚决贯彻党中央国务院、省委省政府和市委决策部署，落实全面从严治党主体责任，履行全面建成小康社会神圣使命。必须坚持贯彻落实科学发展理念，紧密结合实际，创新发展思路，着力富民强市，统筹推进经济发展、社会建设、民生改善、生态建设，实现更有效率、更有质量、更加公平、更可持续发展。必须坚持发展第一要务，始终扭住经济建设中心不动摇，把无中生有抓项目、有中生优增效益作为科学发展的基本方法，夯实发展基础，厚植发展优势，不断开创武威发展新境界。必须坚持改革创新，积极适应经济发展新常态，研究新情况，把握新形势，培育新动能，搭建新平台，落实新要求，以改革创新精神不断推进发展。必须坚持以人民为中心的发展思想，把保障和改善民生作为全部工作的最终目的，发展成果由人民共享，不断增加人民群众的获得感和幸福感。必须坚持依法治市，把政府活动全面纳入法治轨道，依法行政，依法履职，运用法治思维和法治方式推动发展，转作风、强素质、提效率，为转型升级、富民强市提供有力保障。

我们清醒认识到，武威发展从实力聚积到质量提升还有差距，还面临不少困难和问题。经济总量小、人均水平低，效益不高和质量不优的问题突出；工业化程度低，产业链条短，骨干企业和名优品牌少，市场竞争力弱；财政收入总量小与公共服务、民生保障等刚性支出增加的矛盾依然突出；城镇化水平低，城镇带动能力弱，农村基础设施和公共服务体系不完善，改善城乡居住条件等工作还有差距，贫困地区精准脱贫任务紧迫；自然条件严酷、生态环境脆弱的状况仍未根本改变，生态建设保护任重道远；改革开放水平低，一些深层次矛盾和问题还未有效破解；一些公职人员责任意识不强、担当精神不足、真抓实干作风不硬，消极腐败现象在一些领域仍时有发生。我们要正视困难和问题，切实加以解决，决不辜负人民期望。

2017年主要工作

市第四次党代会绘就了未来五年全市发展的宏伟蓝图，确定了经济社会发展的指导思想和主要奋斗目标，对坚决全面从严治党、坚持发展第一要务、努力全面建成小康社会作出了部署。2017年政府工作的总体要求是：全面贯彻党的十八大和十八届三中、四中、五中、六中全会精神，深入学习贯彻习近平总书记系列重要讲话精神和治国理政新理念新思想新战略，按照市第四次党代会部署和全市“十三五”发展规划，坚决全面从严治党、坚持发展第一要务，坚持创新、协调、绿色、开放、共享发展理念，坚持以经济建设为中心、以人民为中心的发展思想。以党建统揽全局、用发展凝聚人心，无中生有抓项目、有中生优增效益，严格标准选干部、不拘一格用人才。大力推进创新驱动，深入实施开放外向、城乡融合、生态立市、工业强市、人才兴市战略。以提高发展质量和效益为中心，促进经济保持中高速增长、产业迈向中高端水平，推动经济总量上台阶、质量效益上水平，强化发展基础，壮大综合实力。坚持经济发展与环境、资源、安全相协调，与社会事业发展同步，与持续增加城乡居民收入并举，统筹推进经济、政治、文化、社会、生态文明建设和党的建设。决战决胜脱贫攻坚，加快建设人民富裕、经济繁荣、生态良好、政治清明、社会和谐的幸福美好新武威，奋力推进全面建成小康社会。

经济社会发展的主要预期目标是：生产总值增长9%，工业增加值增长10%。全社会固定资产投资增长12%，其中规模以上增长10%。一般公共预算收入增长13%。社会消费品零售总额增长11%。城乡居民人均可支配收入增长10%。居民消费价格指数涨幅控制在3%以内。城镇化率提高3个百分点。单位生产总值能耗和主要污染物排放量完成省政府下达目标。

实现上述目标，重点抓好十个方面的工作。

一、全力建设甘肃（武威）国际陆港，打造外向型经济发展平台。全面实施甘肃（武威）国际陆港发展战略规划，建成武威天马机场、城港快速，加快建设陆港中心区道路、供排水工程及综合管廊、武威保税物流中心铁路专用线及集装箱作业区、铁路综合性货场，开工建设陆港110千伏变电站。完成陆港170平方公里外围防护林带及港区廊道绿化，造林5866公顷。争取获批建设武威铁路口岸，申报设立航空口岸和粮食、果品、整车内陆进口等口岸，争取中欧国际货运班列“天马号”开通欧洲国家主要港口城市。着力打造木材产业集聚区和木材集散中心、内陆地区进口肉类产品集散地，构建以现代制造业、进出口加工业、生产服务业为主导，以出口示范农业、文化旅游业为特色的开放外向性产业体系。甘肃路斯宠物食品、陕西天诚家具产业园、武威众兴菌业循环经济示范园等项目建成投产，争取红星美凯龙国际家居产业园、广东宜华家具产业园、中泰工业园、碧桂园、台湾亿光LED光电产业园等项目落地实施。搭建服务平台，扩大产品出口。建成出口农产品基地80万亩，辐射带动周边区域面积150万亩。加快推进陆港周边区建设。

二、大力推进精准扶贫，打好脱贫攻坚战。持之以恒走好“建棚子、栽林子、抓票子、盖房子、过上好日子”的扶贫开发路子，全面落实“1+17+2”规划方案，以小康标准建设贫困区基础设施、培育产业、优化公共服务。全年减贫3.18万人，天祝县整体脱贫。全面完成“下山入川”工程，对生活在自然条件恶劣、缺乏基本生存条件的农牧民应搬尽搬，按照新农村社区规划建设移民安置区，计划移民搬迁23352人。加快发展富民产业，全面落实户均2亩棚（天祝县户均3亩）、人均1亩经济林（天祝县除外）产业发展目标，推进贫困区主体生产模式提质增效，积极发展甜高粱、旱作马铃薯、中药材等优势特色产业，确保贫困人口人均可支配收入增速高于全市平均水平。持续改善基础设施条件，加快推进移民区和贫困村整村建设，实施撤并建制村道路通畅工程、“千村美丽”示范村村组道路硬化工程、公路生命安全防护工程，加快贫困区宽带光缆入村、入户，全面完成建档立卡贫困户危房改建任务，新建一批综合性文化服务中心（乡村舞台）、标准化卫生室。加强扶贫资金监管，对资金使用、低保发放、工程建设中“动手脚”“玩猫腻”的，严厉查处问责，决不手软。

三、加大项目建设和招商引资力度，持续扩大经济总量。深入实施“3341”项目工程，力争实施500万元以上项目600项以上，确保在建项目投资规模保持在1000亿元以上。持续营造“能赚钱、不受气、条件好”的投资环境。依托国际陆港、工业园区、城镇建设大平台，加大招商引资力度，落实到位资金1000亿元以上。北（山）仙（米寺）高速及凉州绕城高速和大靖至干城、凉州至古浪、民勤大滩至南湖、古浪干城至天祝西沟、金色大道至白塔寺景区等公路建成通车，新建、改建农村公路800公里。积极争取兰（州）张（掖）高速铁路早日获批建设。完成红崖山水库加高扩建工程和民勤红砂岗工业集聚区供排水工程，实施凉州区南营、古浪县大靖峡等水库清淤保源工程和病险水库除险加固工程，加快引黄济石精准扶贫生态移民暨沙漠治理供水工程前期工作。加快实施农村饮水安全巩固提升工程，大力推进河西走廊国家级高效节水灌溉示范区建设，发展高效节水灌溉面积20万亩。甘电投武威热电联产项目建成投运，建成古浪40兆瓦光伏扶贫电站、武威热电330千伏配套送出工程和农网改造升级工程，加快天祝松山滩百万千瓦级风电基地建设。围绕产业布局和发展重点，加强项目谋划和储备，做深做实前期工作，改进协调服务，推进项目建设。

四、完善新型工业体系，加快工业经济发展。坚持抓创新、引项目、建园区、上规模、创品牌、增效益，着力培育壮大新能源、新材料、高端装备制造、生物、信息技术、节能环保等战略性新兴产业，打造精细化工、风光电装备制造、碳化硅新材料、核技术民用装备制造、农产品精深加工、液体经济、葡萄酒、制药、家具、包装等产业联盟、产业集群和产业基地。加快民勤红砂岗加氢暨电石精细化工智慧产业园建设，开工建设天祝高性能碳基新材料特色产业基地、北京神雾集团长焰煤节能环保综合利用一体化等项目，遍净集团医药中间体、荣华30万吨鲜奶暨2万吨婴幼儿奶粉等项目建成投产。加大支持工业项目建设政策措施落实力度，新增规上企业40户以上。完善产业园区功能，提升产业集聚发展能力。加快科技合作和成果转化应用，创建省、市级工程技术研究中心和企业技术中心18家。加强工业经济运行调度。严格落实工业节能降耗目标。推进“宽带中国”示范城市建设。

五、持续推进主体生产模式提质增效，提升现代农业发展水平。坚持区域化布局、标准化生产、精细化管理、产业化经营，突出绿色有机，加强设施农牧业标准化建设和特色林果业规范化管理，加快推广新优品种和先进适用技术，建设现代农业信息化服务平台，加强动植物疫病防控，全面提升主体生产模式综合效益。新建设施农牧业5万亩，累计达到95万亩、户均2.8亩、亩均收入2万元；新建特色林果基地10万亩，累计达到178.5万亩、亩均收入5000元。大力推进“贮藏加工+运输销售”现代营销模式，加快培育、引进农产品精深加工、运销企业，推广订单式设施农牧业，加强农产品冷链物流和市场体系建设，加快发展农产品电子商务。严格执行出口农产品生产技术规程，完善农产品质量安全追溯体系，加强质量安全监管，打造一批农产品知名品牌。推进甜高粱规模化种植及精深加工，力争种植面积达到50万亩，引进实施一批下游产品开发项目。

六、加快培育现代服务业体系，促进消费升级。以甘肃（武威）国际陆港为主平台，构建现代物流配送体系和集散中心，培育现代物流企业和物流基地。加快推进红星美凯龙全球家居广场、万嘉汇•台湾士林不夜城、天马中心、天一财富广场、凯帝斯广场、西部荣宝双创园等项目建设，开工建设奇柯奥特莱斯全球商品直销中心等项目，提升黄羊公铁联运物流中心、昊天农产品交易市场、万嘉国际广场运营水平，规划建设一批专业批发市场，改造提升一批蔬菜、畜产品、日用品市场。加快推进全国电子商务进农村综合示范县、省级电子商务示范县和电商扶贫试点县区、乡（村）建设，实施“互联网+”行动，推进云计算、物联网、互联网金融与传统服务业融合发展。加快发展商贸会展、健康养老、文化创意、旅游休闲、体育健身等新兴业态，倡导绿色消费。完善旅游基础设施，强化配套服务功能，全面实施金色大道•马踏飞燕大景区规划和天祝民族风情山水生态文化游规划，加快推进武威丝绸之路文化遗产博览城等文化产业项目和文庙、古钟楼、罗什寺等历史文化街区建设，开工建设海藏湖文化旅游综合体等项目。努力打造丝绸之路黄金旅游带亮点城市和世界知名、中国一流的葡萄酒文化休闲度假地。

七、深入推进城乡融合，加快新型城镇化进程。坚持工业新型化、城镇带状化、农业现代化、社会信息化，全面提升城乡融合发展核心区集聚效应和辐射带动力。以武威城乡融合发展核心区总体规划为统领，实施武威市城市总体规划、土地利用总体规划、历史文化名城保护规划、新型城镇化规划，修编完善县城、工业园区、小城镇、新农村社区和村庄规划，编制综合交通、市政基础设施、公共服务设施、市场体系建设等专项规划。实施中小城市基础设施网络完善工程、县城和重点镇基础设施提升工程，加快凉州城区、县城、重点镇和工业园区、新农村社区道路、供排水管网、集中供热、污水处理、垃圾无害化处理等基础设施建设，稳步推进地下综合管廊建设，推动基础设施和公共服务设施向农村延伸。贯彻“大气灵动、兴业宜居”理念，配套完善凉州城区功能。加强凉州城区和县城、重点乡镇市容环境综合整治。加快天马湖东区、海藏湿地公园建设。推进智慧城市建设。改善城乡居民住房条件，继续实施公租房项目，改造棚户区12000户，加快保障房分配入住。规划建设G30、金色大道两侧新农村社区，新建、续建新农村社区40个。加快民勤、天祝县城和清源葡萄酒小镇、黄羊川镇、炭山岭镇、红砂岗镇等特色小镇建设。积极创建省级“千村美丽”示范村和“万村整洁”示范村，大力整治农村环境卫生，推进美丽宜居乡村建设。

八、加强生态保护建设，促进生态环境持续好转。坚持抓好“节水、治沙、造林、防污”四个重点，完善“国家有投入、企业给赞助、科技作支撑、农民有收益”的生态建设长效机制，推进压沙修路、平整沙丘、造林绿化、工业治沙、生态农业等治沙和沙产业发展模式，实施山水林田湖生态保护和修复工程，加快建设全省生态环境保护示范区、全国防沙治沙综合示范区和生态文明示范区。坚持以水定产业、以水定结构，压减农业用水、节约生活用水、增加生态用水、保证工业用水，全面推进节水型社会建设。巩固扩大石羊河流域重点治理成果，谋划实施后续治理项目，争取加大外流域调水力度。继续实施人工增雨雪。加快推进千里沙漠大林带工程、甘肃建投工程与生物治沙、三北防护林、新一轮退耕还林还草、濒危野生动物民勤青土湖试验性放养基地等重点项目，加强石羊河国家湿地公园和青土湖、黄案滩等荒漠湿地建设，强力推进“下山入川”移民迁出区、祁连山保护区建设项目整治区生态恢复，禁止祁连山保护区核心区人类活动。抓好国际陆港、武威天马机场、北（山）仙（米寺）高速、G338武威段、“下山入川”移民区等重点区域绿化造林。严格执行“五禁”决定。坚持干部义务压沙造林制度。加强治沙生态林承包经营管理。完成人工造林45万亩，封山（沙）育林（草）30万亩。实行最严格的环境保护制度，加强环境执法，健全环境监管体制，实施大气、水、土壤污染防治行动计划，治理环境噪音污染，持续打好凉州城区大气污染防治攻坚战，从严控制工业点源、农业面源和城乡生产生活污染，全面完成省上下达的环境质量改善和总量减排目标任务。

九、协调发展社会事业，提高人民群众生活水平。大力支持大众创业、万众创新，落实更加积极的就业创业政策，城镇新增就业2.2万人以上，高校毕业生就业率达到85%以上，城镇登记失业率控制在4%以内。加大城乡居民养老保险和被征地农民社会保障政策落实力度，加强养老基础设施和服务体系建设。对有集中供养意愿、完全丧失劳动能力的残疾人全部实现集中供养。推进城乡居民基本医疗保险制度整合，全面实施城乡居民大病保险。扩大住房公积金制度覆盖面。推进义务教育均衡发展，实施“全面改薄”项目，优化教育资源配置，确保学前教育三年毛入园率达到80%、九年义务教育巩固率保持在99.5%以上、高中阶段教育毛入学率达到94%以上。加快实施武威一中迁建提升工程，推进天祝民族中学、古浪一中、武威十八中创建省级示范性普通高中。加快高等教育发展，创建新能源化工大学、常州大学武威工学院，支持武威职业学院加快发展。建立全民健康档案，2017年建成45万人（份）。加快市人民医院整体搬迁二期工程、武威职业学院直属附属医院建设，武威重离子治疗肿瘤中心暨荣华颐养园建成投运。精心实施恶性肿瘤防控工程。全面推进县级公立医院综合改革。完善落实二孩政策，促进人口均衡发展。完善公共文化体育服务设施，博物馆、影剧院、图书档案馆、城乡融合规划馆投入运行。建成乡镇及社区体育健身中心、村级体育场127个。贯彻普惠性、保基本、均等化、可持续要求，在教育、卫生、健康、公共交通等方面为人民群众办一批实事。加强精神文明建设，引导城乡居民更新观念，讲文明、改陋习、树新风。加强安全生产工作，严格落实安全生产政府领导责任、部门监管责任、企业主体责任，排查治理安全隐患，推进巡查常态化，严肃执法和问责，保持安全生产形势稳定。实行最严格的食品药品安全监管，确保人民群众饮食用药安全。健全突发公共事件预警、灾害应急救助和防灾减灾体系。深化平安武威建设，创新社会治理，建设社会治安防控体系，整治社会治安重点区域和突出问题，依法严厉打击各类违法犯罪活动。积极推进军民融合，密切军政军民关系。深入开展民族团结进步创建活动，推进天祝县“两个共同”示范县建设，依法加强民族宗教事务管理，确保社会大局和谐稳定。

十、全面深化改革，增强发展动力。加快推进供给侧结构性改革，全面落实降低实体经济企业成本政策措施，提升企业竞争力。调整优化房地产供给结构，增加高端有效供给，消化低端存量，促进房地产市场健康发展。积极推行政府与社会资本合作（PPP）模式，支持社会资本进入特许经营领域，扩大参与基础设施项目、市政公用事业建设和运营。推进创新驱动，加强自主创新、科技创新、管理创新，激发创新活力。深化商事制度改革，落实公平市场准入、公平资源配置、拓展投资领域、支持自主创新等改革措施。全面完成不动产登记信息管理平台建设。深化户籍制度改革，全面实行居住证制度。推进价格机制改革，进一步减少政府定价项目，实现竞争性领域和环节的价格基本放开。全面推进水价综合改革。加快推进电力价格市场化改革。深化财税体制改革，促进公共财政预算收入持续稳定增长。推进投融资体制改革，实施“引行入武、推行下县”行动，组建古浪县、天祝县农商银行，鼓励企业上市融资或到股权交易市场挂牌融资，加大对重点产业、项目和“三农”信贷支持力度，切实解决中小微企业融资难、融资贵问题，有序推进农村“三权”抵押贷款，加强社会信用体系建设，维护金融生态环境。深化农村集体产权制度改革，积极推进集体林权制度配套改革、国有林场改革，加快供销合作社综合改革。深化“放管服”改革，再清理取消下放调整一批行政审批事项，全面推行“双随机一公开”监管改革，清理规范中介服务。深化政务公开，优化政府服务。严格机构编制管控，财政供养人员只减不增。

各位代表，全面贯彻落实市第四次党代会精神，完成2017年经济社会发展目标任务，对政府自身建设提出了更高更严的要求。我们要加快转变政府职能，创新管理服务方式，不断提升政府工作的创造力、执行力和公信力。

一是坚持理论武装，建设学习型政府。始终保持能力危机感和本领恐慌感，深入学习习近平总书记系列重要讲话精神和治国理政新理念新思想新战略，全面贯彻执行党的基本路线，学习新政策，掌握新知识，增强新本领，准确把握经济形势和发展规律，提高适应经济发展新常态、创造性贯彻重大决策部署和抢抓机遇、谋划发展、推动落实的能力。

二是坚持解放思想，建设创新型政府。坚持以创新引领发展，着力推进理念创新、政策创新、机制创新和方法创新，提升创新能力，用新理念指导实践，用新思维破解难题，用新举措推动发展。完善和落实更加开放、灵活、务实、管用的人才政策，加大急需紧缺人才和高层次人才引进培养力度，推进人才高地建设，为创新发展提供人才支撑。

三是坚持依法行政，建设法治型政府。落实法治政府建设实施方案，全面推进依法治市，加快建设法治武威。严格按照法定权限和程序履行职责，坚持依法决策、依法行政。深入开展“七五”普法，推动全社会尊法、学法、守法、用法。完善政府权力清单、责任清单，自觉接受市人大及其常委会的法律监督和工作监督，自觉接受市政协的民主监督，自觉接受社会舆论和人民群众的监督，认真办理人大代表建议、政协委员提案，及时回应人民群众关切，推进权力依法公开透明运行。

四是坚持履职为民，建设服务型政府。坚持全心全意为人民服务的根本宗旨，坚持发展为了人民、发展成果由人民共享，聚精会神抓发展、强实力，重民生、惠民利。坚持问政于民、问需于民、问计于民，努力使政府作出的决策部署、出台的政策措施更加符合人民群众期待，政府工作更加切合人民群众要求，公共资源更多向民生倾斜。脚踏实地为群众办实事、解难事，给予困难群众更多关心和帮助，当好人民公仆，让人民群众过上更加幸福美好的生活。

五是坚持真抓实干，建设责任型政府。坚持问题导向，在解决问题中推进工作落实。着力提升效能，坚决整治不作为、慢作为、怕担当、不负责等庸政懒政怠政问题，警惕伪善伪廉伪勤问题。加强督促检查，切实解决重安排、轻落实，有令不行、有禁不止，推诿扯皮、敷衍塞责等问题。坚持抓严抓紧抓细抓实，走在前列、干在实处，恪尽职责、善做善成，体现人民政府高度的责任担当。

六是坚持勤政廉政，建设廉洁型政府。模范遵守党章党规，严守党的政治纪律和政治规矩，严格落实全面从严治党责任。认真学习贯彻《关于新形势下党内政治生活的若干准则》，加强和规范党内政治生活，坚持民主集中制，落实党的组织生活各项制度，增强党内政治生活的政治性、时代性、原则性、战斗性。严格执行中国共产党《廉洁自律准则》《纪律处分条例》《问责条例》《党内监督条例》和中央八项规定、省委、市委“双十条”规定，讲规矩、守纪律、严监督，做到忠诚干净担当。完善权力运行制约和监督机制，加强行政监察和审计监督，有权必有责、用权必担责、滥权必追责。坚持把纪律和规矩挺在前面，有腐必反、有贪必肃，严肃查处腐败案件，增强拒腐防变和抵御风险能力，构建不敢腐、不能腐、不想腐的有效机制，推进干部清正、政府清廉、政治清明。

各位代表，实干收获希望，奋斗创造未来。我们要更加紧密地团结在以习近平同志为核心的党中央周围，在市委的坚强领导和市人大、市政协的监督支持下，团结依靠全市各族人民，不忘初心、继续前进，担当尽责、奋发进取，为建设幸福美好新武威、全面建成小康社会而努力奋斗！
